# Leptospirosis in Taiwan, 2001–2006

**DOI:** 10.3201/eid1405.070940

**Published:** 2008-05

**Authors:** Yu-Ling Chou, Chang-Shun Chen, Cheng-Chung Liu

**Affiliations:** * Centers for Disease Control, Taipei, Taiwan, Republic of China; † Institute of Plant and Microbial Biology, Taipei, Taiwan, Republic of China

**Keywords:** Leptospirosis, incidence, climate, rodent, rainfall, letter

**To the Editor:** Leptospirosis is a zoonotic disease that has now been identified as an emerging infectious disease (*1*,*2*). It is caused by pathogenic spirochetes of the genus *Leptospira*. The natural hosts for *Leptospira* spp. come from a variety of species (*2*–*4*), of which the rodent is the most important reservoir (*4*,*5*). The incubation period range for leptospirosis is usually 5–14 days, with a range of 2–30 days (*4*). Leptospirosis is a disease of humid tropical and subtropical countries. According to the World Health Organization (*4*), probable leptospirosis incidence ranges from ≈0.1–1 case/100,000 population/year in temperate climates to 10–100 cases/100,000 population/year in humid tropical climates. Leptospirosis epidemics are often related to heavy rainfall and flooding (*1*,*6*,*7*). Because of its climate, Taiwan may be at high risk for leptospirosis. We therefore investigated human leptospirosis in Taiwan and the relationship between leptospirosis incidence and rainfall pattern.

Taiwan is a medium-sized archipelago in East Asia; the Tropic of Cancer runs through its center. The northern part of Taiwan is subtropical; the southern part is tropical. Taiwan lies in the path of many tropical storms and typhoons that bring extremely heavy rainfall usually during July–September. The annual “plum rain” season in May and June also brings a lot of precipitation. Because of its tropical and subtropical marine climate, Taiwan enjoys rich agricultural productivity throughout the year, which is favorable for rodent infestations (*8*,*9*).

In Taiwan, reported cases of leptospirosis have been investigated by the Centers for Disease Control since 2001. Leptospirosis should be suspected in patients who have fever; headache; myalgia; abdominal pain; prostration; conjunctival suffusion; meningeal irritation and aseptic meningitis; anuria, oliguria, or proteinuria; jaundice; acute renal insufficiency; or gastrointestinal or lung hemorrhage. Patients with suspected leptospirosis are reported by physicians to Taiwan’s Centers for Disease Control through the Notifiable Disease Surveillance System, after which local health bureaus collect urine and blood samples for confirmation by serologic testing. Urine and blood samples from patients with clinically suspected leptospirosis are inoculated into Ellinghausen-McCullough-Johnson-Harris culture medium plus 5-fluorouracil and incubated at 30°C for 8–12 weeks. Cultures are examined by dark-field microscopy every week. Alternatively, latex agglutination assay may be used for rapid serologic diagnosis of serum from patients with clinically suspected leptospirosis (*10*). Samples with positive latex agglutination assay results should be confirmed by microscopic agglutination test (MAT). An antibody titer >100 as determined by MAT is regarded as a probable case of leptospirosis. The local health bureau again collects patients’ serum during the convalescent phase of illness for confirmation by MAT. A laboratory-confirmed case is defined as the isolation of leptospires from urine and blood or a 4-fold increase in antibody titer between acute- and convalescent-phase samples.

During 2001–2006, of 7,733 suspected human cases of leptospirosis, 291 cases were confirmed. The major serotype identified was *L. santarosai* serovar Shermani. The mean annual incidence was 0.21 cases/100,000 population. The laboratory-confirmed cases were observed in Taiwan, mostly in male patients (83.5%) (Figure, **panel A**). Cases occurred in all age groups but were more common (90%) in those 25–74 years of age. Age-specific incidence was highest for persons 55–64 years of age; mean annual incidence was 0.57/100,000 population.

**Figure:**
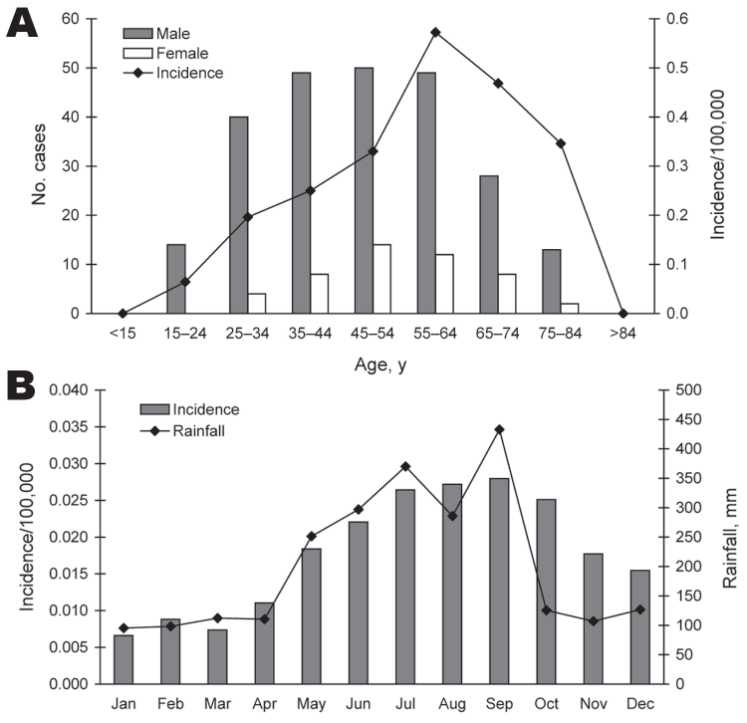
Leptospirosis cases (N = 291) in Taiwan, 2001–2006. A) Distribution by age and sex. B) Relationship between rainfall and leptospirosis incidence. Data represent averages for each month during the 6 years.

Rainfall data from the Central Weather Bureau of the Republic of China showed typically high rainfall (252–433 mm/month) in Taiwan during May–September. Heavy rains were followed by an increase in laboratory-confirmed cases of leptospirosis (Figure, **panel B**); June–October accounted for 60% of cases, with a higher incidence of 0.022–0.028 cases/100,000 population. In October–December, monthly rainfall was below average (201.1 mm/month), but leptospirosis incidence was above average (0.018/100,000). Specifically, 25 (74%) of leptospirosis cases in October, 10 (42%) in November, and 4 (19%) in December were likely associated with several days of heavy rainfall from typhoons. Therefore, the typhoons may be the reason for high incidence in October–December.

 The annual incidence of leptospirosis in Taiwan is relatively lower than that in other countries with tropical or subtropical climates. Our study does not conclusively document the reason for lower incidence, although it does suggest an association between amount of rainfall and incidence. An understanding of the relationship between leptospirosis incidence and rainfall is indispensable for implementing appropriate preventive measures.
